# Local factors associated with on‐host flea distributions on prairie dog colonies

**DOI:** 10.1002/ece3.4390

**Published:** 2018-08-14

**Authors:** Robin E. Russell, Rachel C. Abbott, Daniel W. Tripp, Tonie E. Rocke

**Affiliations:** ^1^ U.S. Geological Survey National Wildlife Health Center Madison Wisconsin; ^2^ Colorado Division of Parks and Wildlife Wildlife Health Program Fort Collins Colorado

**Keywords:** climate, *Cynomys* spp., flea, plague, prairie dog, *Yersinia pestis*

## Abstract

Outbreaks of plague, a flea‐vectored bacterial disease, occur periodically in prairie dog populations in the western United States. In order to understand the conditions that are conducive to plague outbreaks and potentially predict spatial and temporal variations in risk, it is important to understand the factors associated with flea abundance and distribution that may lead to plague outbreaks. We collected and identified 20,041 fleas from 6,542 individual prairie dogs of four different species over a 4‐year period along a latitudinal gradient from Texas to Montana. We assessed local climate and other factors associated with flea prevalence and abundance, as well as the incidence of plague outbreaks. *Oropsylla hirsuta*, a prairie dog specialist flea, and *Pulex simulans,* a generalist flea species, were the most common fleas found on our pairs. High elevation pairs in Wyoming and Utah had distinct flea communities compared with the rest of the study pairs. The incidence of prairie dogs with *Yersinia pestis* detections in fleas was low (*n* = 64 prairie dogs with positive fleas out of 5,024 samples from 4,218 individual prairie dogs). The results of our regression models indicate that many factors are associated with the presence of fleas. In general, flea abundance (number of fleas on hosts) is higher during plague outbreaks, lower when prairie dogs are more abundant, and reaches peak levels when climate and weather variables are at intermediate levels. Changing climate conditions will likely affect aspects of both flea and host communities, including population densities and species composition, which may lead to changes in plague dynamics. Our results support the hypothesis that local conditions, including host, vector, and environmental factors, influence the likelihood of plague outbreaks, and that predicting changes to plague dynamics under climate change scenarios will have to consider both host and vector responses to local factors.

## 1 INTRODUCTION

Sylvatic plague, caused by the bacteria *Yersinia pestis*, manifests in the western United States as episodic outbreaks in prairie dogs, squirrels, and small rodents, with occasional spillover to human populations (Gage, 1998). Prairie dogs (*Cynomys* spp.) are highly susceptible to plague and colonies often suffer >90% mortality during plague outbreaks (Cully & Williams, 2001). By decimating prairie dog populations, plague can have devastating effects on species such as black‐footed ferrets (*Mustela nigripes*), which depend on prairie dogs for their prey (Antolin et al., 2002). At present, the factors driving plague outbreaks are difficult to ascertain. A number of studies have indicated that small rodent composition (Stapp et al., 2009) and local weather conditions (Collinge et al., 2005; Hubbart, Jachowski, & Eads, 2011; Savage, Reich, Hartley, Stapp, & Antolin, 2011) may play a role in contributing to plague epizootics, potentially through their effects on flea distribution and abundance. However, these factors likely vary geographically in the strength of their effects. As the primary vector of plague, fleas play an important role in the dynamics of the disease (Bacot & Martin, 1914). In order to understand the conditions that are conducive to plague outbreaks and potentially predict spatial and temporal variations in risk, it is important to understand the factors associated with flea abundance, prevalence, and distribution that may lead to plague outbreaks.

Traditionally, the hypothesized mechanism for flea transmission of plague bacteria was the result of “blocking” (Bacot & Martin, 1914), whereby *Y. pestis* bacteria form a blockage in the midgut of fleas, causing them to increase the number of feeding attempts and regurgitate infectious material, increasing the likelihood of plague transmission. Lorange, Race, Sebbane, and Hinnebusch (2005) developed models that suggested, with this mechanism of transmission, high flea abundance was necessary for driving plague epizootics due to the low competence of fleas as vectors and the short lifespan of blocked fleas. Eisen et al. (2006) introduced the idea of early‐phase transmission to explain the rapid spread of plague through host populations, as an alternative hypothesis to blocking. Evidence of early‐phase transmission, which does not require blocking of fleas, was observed in *Oropsylla montana* (Eisen et al., 2006), and modeling has demonstrated that early‐phase transmission can drive plague epizootics (Buhnerkempe et al., 2011). Previous research has demonstrated that the ability of fleas to block decreases at higher temperatures (Cavanaugh, 1971; Hinnebusch, Fischer, & Schwan, 1998; Kartman, 1969), therefore potentially explaining the relationship between decreased temperature and frequency of plague outbreaks (Cavanaugh, 1971; Collinge et al., 2005; Enscore et al., 2002; Pollitzer, 1954). However, in contrast, early‐phase transmission in *Xenopsylla cheopis* was delayed or inhibited at cooler temperatures, but did not seem to be affected by warmer temperatures (Schotthoefer et al., 2011). These results indicate that local climate conditions may affect flea biology and the likelihood of plague outbreaks in prairie ecosystems.

Numerous factors likely affect on‐host flea prevalence (number of hosts with fleas) and abundance (number of fleas on hosts). Previous research has indicated that on‐host flea abundance has been associated with local weather conditions, although the relationships are seemingly complex. Increasing flea abundance has been associated with dry summers in a New Mexico population of black‐tailed prairie dogs (BTPD, *C. ludovicianus*; Eads, Biggins, Long, Gage, & Antolin, 2016), wetter springs and cooler summers on great gerbils (*Rhombomys opimus*) in Kazakhstan (Stenseth et al., 2006), and warmer autumns in the previous year in Mongolian gerbils (*Meriones unguiculatus*; Xu et al., 2015), while decreasing flea abundance was associated with increasing prior year growing season and winter precipitation in South Dakota BTPD (Eads & Hoogland, 2016).

Several studies have examined the relationship between the incidence of plague and climatic variables. Some studies have proposed that hot weather desiccates flea larvae and reduces overall flea abundance (Snäll, O'Hara, Ray, & Collinge, 2008), therefore, reducing the likelihood of plague, while others have proposed that drier weather leaves hosts in poorer condition resulting in increased flea abundance and increased potential for plague outbreaks (Eads et al., 2016). Precipitation also may affect the likelihood of plague, with increased precipitation leading to better forage conditions and increased host densities, which may create favorable conditions for plague outbreaks (Enscore et al., 2002). For example, Stenseth et al. (2006) found that the incidence of *Y. pestis* in great gerbils in Central Asia increased with warmer springs and wetter summers. Eads and Hoogland (2017) observed that flea abundance on Gunnison's prairie dogs (*C. gunnisoni,* GPD) in Arizona and white‐tailed prairie dogs (*C. leucurus*, WTPD) in Colorado varied inversely with increasing precipitation during the prior year growing season and suggested that this may explain increased risk of plague after drought. Enscore et al. (2002) found that the numbers of human plague cases in New Mexico and Arizona decreased with increasing summer temperatures and increased when spring precipitation in previous years was high. However, greater summer rainfall, but not previous year precipitation, was associated with plague events in BTPD in Colorado (Savage et al., 2011). At last, Eisen et al. (2012) suggested that flea diversity, which was positively related to rainfall and negatively related to temperature, may play a role in plague outbreaks in Uganda.

In addition to climate and weather variables, flea and host characteristics may influence flea abundance. *Oropsylla hirsuta* and *O. tuberculata*, flea species strongly associated with prairie dog colonies and plague outbreaks, exhibit seasonal trends (Mize & Britten, 2016). The abundance of *O. tuberculata* is often highest in early spring (Cully, Barnes, Quan, & Maupin, 1997; Salkeld & Stapp, 2008; Tripp, Gage, Montenieri, & Antolin, 2009), while *O. hirsuta* numbers tend to peak in mid or late summer (Salkeld & Stapp, 2008; Tripp et al., 2009). Eads et al. (2013) noted that overall prevalence of fleas also fluctuated seasonally. Flea abundance on BTPD has been found to be higher on adult males than on juvenile and/or adult female prairie dogs in Colorado (Tripp et al., 2009). However, in South Dakota, no difference in flea abundance was found on male and female BTPD, although juveniles had fewer fleas than adults (Eads & Hoogland, 2016).

In summary, the relationships between weather factors, flea abundance, and host characteristics (body condition or abundance) remain difficult to ascertain and may not be consistent between different host species, fleas, and locations. We collected fleas from four species of prairie dogs over a 4‐year period (2013–2016) on paired plots located along a latitudinal gradient from Montana to Texas and assessed local climate and other factors associated with flea prevalence and abundance (total fleas, *O. hirsuta* and *P. simulans* counts), as well as the incidence of plague outbreaks. Elucidating the relationship between flea species composition, abundance and distribution, the occurrence of plague, and environmental factors will provide information to management agencies responsible for controlling plague, as well as help predict how plague dynamics may change under future climate conditions.

## 2 METHODS

### 2.1 Study areas and design

**Figure 1:**
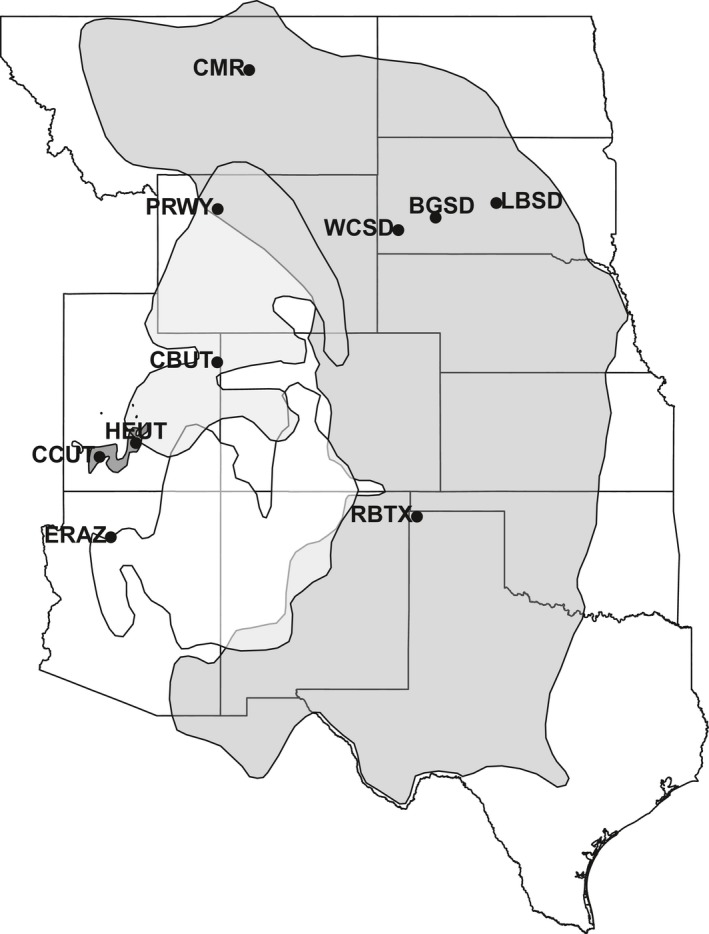
Map of study pairs. Polygons indicate the ranges of different prairie dog species; medium gray for black‐tailed prairie dogs (CMR: Charles M. Russell, Montana; WCSD: Wind Cave, South Dakota; BGSD: Buffalo Gap, South Dakota; LBSD: Lower Brule, South Dakota; RBTX: Rita Blanca, Texas), light gray for white‐tailed prairie dogs (PRWY: Pitchfork Ranch, Wyoming; CBUT: Coyote Basin, Utah), dark gray for Utah prairie dogs (CCUT: Cedar City, Utah; HEUT: High elevation, Utah), and white for Gunnison's prairie dogs (ERAZ: Espee Ranch, Arizona)

We analyzed flea abundance and prevalence on prairie dogs from 23 paired plots that had been included in a large‐scale sylvatic plague vaccine (SPV) trial (see further details in Rocke et al., 2017). A pair consisted of one plot treated with SPV‐laden baits and one plot that received placebo baits. In brief, our study included 11 paired plots on BTPD colonies, one pair on a GPD colony, four pairs on WTPD, and seven pairs on Utah prairie dog (UPD, *Cynomys parvidens*) colonies, sampled over a 3‐year period, 2013–2015 (Figure 1). Fifteen of the pairs were resampled in 2016. For plots to be included in the study, we required that no pesticides had been used for at least one year prior to the beginning of the study. Most pairs had never been dusted; however, two pairs (4 plots) (Charles M Russell, Montana‐CMR) were dusted in 1997, and one pair (Wind Cave, South Dakota‐WCSD) was dusted in 2011. Tripp et al. (2016) observed a waning effect of dusting on flea abundance on sites in Colorado after 10–12 months.

SPV‐laden baits were distributed on one randomly assigned plot in each pair and placebo baits were distributed on the other. Within 1–4 weeks, prairie dogs were trapped and sampled on the same days at both plots within a pair by the same personnel using Tomahawk live traps. Traps were baited with sweet feed and oats. Sex, age, weight, and foot length were recorded for each animal sampled, and fleas and other samples were collected from each animal upon first capture during a sampling period. Sampling dates varied by year, but occurred between June and November (Appendix 1), and a sampling period consisted of a minimum of 3 days of trapping (on consecutive days unless prevented by field conditions) (Appendix 2). During 2013, prairie dogs were sampled both pre‐ and post‐baiting, with approximately a month between sampling periods. In 2014–2015, each plot was sampled once (with the exception of the GPD pair and one of the WTPD pairs in Utah which were sampled twice, once in July and once in August).

For flea sampling, animals were anesthetized with isoflurane in an induction chamber and immediately combed for fleas that were collected into tubes of sterile saline or ethanol. After sample collection, animals were allowed to fully recover from anesthesia and then released at the location of capture (NWHC Animal Care and Use Committee, Protocol #EP130214). Fleas collected from live prairie dogs were stored at −20°C until identification. To remove surface contamination that might alter PCR results, fleas were rinsed with 70% ethanol containing 0.2% iodine and rinsed twice in sterile water (La Scola, Fournier, Brouqui, & Raoult, 2001; Raoult et al., 2001;  Kernif et al., 2014). Fleas were then counted and identified to species using published taxonomic references (Furman & Catts, 1982; Hubbard, 1947; Stark, 1970), and pooled by species and sex, up to 10 individuals per pool from a single animal.

To determine plague status of our study plots, spleen and liver tissues from prairie dog carcasses, as well as flea pools collected from live and dead animals, were tested for the presence of *Y. pestis* using real‐time PCR (see Rocke et al., 2017 for details). In brief, tissue DNA was extracted using the Wizard SV Genomic DNA Purification System (Promega; Madison, WI), and PCR was performed for the *caf1* gene sequence located on the *Y. pestis* pG8786 plasmid (Genbank accession NC_006323) and the *pla* gene located on the *Y. pestis* pPCP1 plasmid (Genbank accession AL109969). Tissues from carcasses were also cultured on blood agar plates, and suspect colonies were confirmed as *Y. pestis* using real‐time PCR. In the absence of positive cultures, DNA was only considered positive for *Y. pestis* if both the *pla* and *caf1* genes were present. Flea DNA was extracted using the Zymo Quick gDNA Miniprep Kit (>1 flea) or Micro Kit (1 flea) (Zymo Research; Irvine, CA) depending on the number of fleas in each pool. DNA samples were then screened for the *pim* gene, which resides on the pPCP1 plasmid of *Y. pestis*, using real‐time PCR, as we found this gene to be more sensitive than the *pla* gene for fleas. Suspect positive fleas were then confirmed using *caf1* gene as described above. A plot was classified as plague positive if at least one carcass or one flea pool from a live prairie dog tested positive for *Y. pestis* by culture or PCR. Once a plot was classified as “plague positive,” it was considered plague positive in subsequent years.

### 2.2 Statistical analyses

We do not formally account for detection error in our analyses; therefore, we have no repeated measures from which to estimate detection (see Eads et al., 2013 for methods with repeat detections). The amount of time spent combing for fleas was not standardized between pairs (although we assume effort between paired plots was similar); therefore, our counts should be considered relative indices. We used logistic regression and negative binomial regression with LASSO (least absolute shrinkage and selection operator; Tibshirani, 1996) priors in a Bayesian framework to assess factors related to total flea abundance, *O. hirsuta* abundance, and *Pulex simulans* abundance. This allowed us to examine factors related to both the number of fleas on a host (abundance) and the presence/absence of fleas on a host (prevalence). The LASSO allows for model selection and regularization to take place automatically, while improving predictive accuracy and providing interpretable parameter estimates (Tibshirani, 1996). Parameter estimates for variables that are not important predictors shrink to zero. We demonstrate fit of the data to a negative binomial distribution graphically (Appendix 3).

We evaluated factors associated with prevalence of fleas and overall flea abundance, including variables related to climate and seasonal weather patterns (NDVI [normalized difference vegetation index—a measure of vegetation greenness, see Table 1], number of days with a temperature over 85°F [Collinge et al., 2005], amount of precipitation during the prior year's growing season, winter precipitation amount, and day of sampling), plot‐level variables (catch per unit effort as an index of relative abundance of prairie dogs [see Rocke et al., 2017 for details], treatment [placebo vs. vaccine], plague status [plague detected vs. not detected], and elevation), and individual covariates (age, sex, body condition [=weight/foot length]) (Table 1). To incorporate effects of plot, we used a random effect for BTPD (*n* = 22 plots), UPD (*n* = 14), and WTPD (*n* = 8). Three of seven UPD pairs were located on the same prairie dog colony and significant movement was noted between the plots on these pairs, so treatment effects at these pairs may be compromised. GPD were collected at one pair of plots only, therefore, accounting for location was not necessary. For *O. hirsuta* and *P. simulans* abundance, we used the same covariates as described above; however, negative binomial regression was only appropriate for BTPD, due to the relatively few numbers of hosts with >1 flea observed for other species. Therefore, for all other species, we report results for logistic regression only.

**Table 1:** Description of covariates used in statistical analyses of flea abundance

Climate and weather variables	
Day of sampling	Day in the year (1–365) sampling took place
Winter precipitation	Centimeters of precipitation January‐April of current year (from NOAA Annual Climatological Survey, https://www.ncdc.noaa.gov/cdo-web/datatools/findstation)
Number of days > 85°F	Number of days in year with a temperature over 85 degrees F (from NOAA Annual Climatological Survey, https://www.ncdc.noaa.gov/cdo-web/datatools/findstation)
Prior year growing season precipitation	Centimeters of precipitation April‐August of previous year (from NOAA Annual Climatological Survey, https://www.ncdc.noaa.gov/cdo-web/datatools/findstation)
Normalized difference vegetation index (NDVI)	NDVI values from 7‐day Moderate Resolution Imaging Spectroradiometer (MODIS) composites that included the day of sampling for the center point of each plot were extracted for each plot (data available from the U.S. Geological Survey, https://lta.cr.usgs.gov/emodis; Jenkerson, Maiersperger, & Schmidt, 2010)
Plot‐level characteristics	
Elevation	Elevation in meters at center point of plot NED digital elevation map (from https://lta.cr.usgs.gov/NED, Archuleta et al., 2017)
Plague (detected, not detected)	Plague detected or plague not detected
Catch per unit effort (CPUE)	Number of individuals/number of trap days (see Rocke et al., 2017 for more details)
Treatment (vaccine, placebo)	Vaccine or placebo (see Rocke et al., 2017 for more details)
Flea species diversity	Number of flea species detected on a particular plot
Individual Characteristics	
Age (adult, juvenile)	Categorical variable adult = 1, juvenile = 0
Body condition	Body condition: weight in g/foot length in mm
Sex (male, female)	Categorical variable male = 1, female = 0

We estimated factors associated with initial plague detection on placebo plots using logistic regression, including all plot‐level factors previously mentioned and flea species diversity as predictor variables. Data were summarized for each plot by year combination, and because we were interested in determining factors related to initial plague outbreaks (the first year plague was detected in fleas or carcasses), plot by year combinations with continuing plague outbreaks were excluded. We were interested in evaluating conditions that could predict plague outbreaks; therefore, we included catch per unit effort in the previous year. If we had instead related catch per unit effort in the year of initial plague detection rather than the year before, our models would inherently predict that low catch per unit effort is associated with plague outbreaks (i.e., the two variables are confounded). The inclusion of catch per unit effort from the previous year required us to exclude all 2013 data for this analysis, because we did not have prior year data (in any case, no plague was detected on any plots in 2013), leaving 55 plot by year combinations (including 8 initial plague detections on plots).

For all models, logistic and negative binomial, we calculated effect sizes by exponentiating the parameter estimates. Effects estimated from parameters of a negative binomial indicate the change in the expected number of fleas on a host given a 1 unit increase in the covariate (or a change in the covariate from one categorical level to another); we represent these effects as a percent change. Effects for coefficients from a logistic regression are odds ratios and indicate the odds of flea presence given a 1 unit increase in the covariate. Odds ratios of 1 indicate no change in the probability of presence given an associated increase in the covariate value, odds ratios<1 indicate that the probability of presence is less likely given an associated increase in the covariate value, and odds ratios >1 indicate an increase in the probability of presence.

All models were run in R (R Core Team, 2017). Models were run for a total of 60,000 iterations with a burn in of 20,000. All models were run in rjags (Plummer, 2013). We used Gelman–Rubin diagnostics to assess convergence (Gelman & Rubin, 1992). We used area under the curve statistics in the “pROC” package (Xavier et al., 2011) to evaluate goodness of fit for our logistic regression models. For negative binomial models, we used posterior predictive checks on the parameters of the negative binomial distribution by simulating 1,000 data sets from the estimated model parameters and comparing to the observed data (Gelman, Carlin, Stern, & Rubin, 2004). To display a visual representation of flea community structure, we used nonmetric multidimensional scaling (NMDS; an ordination technique) in R (package “vegan”; Oksanen et al., 2017). We assessed the fit of the NMDS by reporting a “stress” value (Oksanen et al., 2017).

## 3 RESULTS

In total, we identified 20,041 fleas from 6,542 prairie dogs (Table 2, Appendix 4) sampled between June and November over a 4‐year period, including 3,526 BTPD, 249 GPD, 1,590 UPD, and 1,177 WTPD. Four hundred and fifteen prairie dogs were sampled twice in the same year; however, samples were collected at least 30 days apart (at pre‐ and post‐bait intervals) and were therefore treated independently. We identified 18 species of fleas. The prairie dog specialist flea *O. hirsuta* was the most common species detected, comprising 59% of all fleas sampled and occurring on all pairs except UPD pairs located at high elevation (HEUT) and both WTPD pairs in Wyoming (PRWY). These pairs were also the most diverse, with 10 flea species detected at HEUT and 6 flea species at PRWY. The second most commonly detected flea species was *Pulex simulans,* a generalist flea species, accounting for 23% of fleas identified. All *Pulex* fleas were assumed to be *P. simulans*, because no male *Pulex irritans* were identified (Tripp et al., 2009). *Pulex simulans* was found mainly on BTPD pairs in Montana and Texas and WTPD pairs in Utah, with sporadic detections elsewhere. The ground squirrel flea *Thrassis francisi* comprised ~10% of detections and was most commonly detected on high elevation pairs in Utah. Three other *Oropsylla* species, *O. tuberculata, O. labis*, and *O. idahoensis*, comprised less than 10% of detections and were most commonly found on pairs where *O. hirsuta* was not detected. Two other species, *Neopsylla inopina* (a ground squirrel flea) and *Aetheca wagneri* (a common flea of *Peromyscus* spp.), were rarely encountered (<2% of detections). *N. inopina* was found only at PRWY, while only 6 *A. wagneri* were detected on BTPD and UPD (Appendix 5) during the entire study. Forty‐five fleas (<1%) of 10 additional species were identified (<20 of each) (Appendix 6).

**Table 2:** Total number of fleas, prairie dogs sampled, and hosts with fleas. Flea prevalence = hosts with fleas/hosts sampled, flea intensity = total fleas/hosts with fleas, mean flea abundance = total fleas/hosts sampled. BTPD is black‐tailed prairie dog (*Cynomys ludovicianus*), GPD is Gunnison's prairie dog (*Cynomys gunnisoni*), UPD is Utah prairie dog (*Cynomys parvidens*), and WTPD is white‐tailed prairie dog (*Cynomys leucurus*)

Study area and species	Acronym	Total fleas	Prairie dogs sampled	Prairie dogs with fleas	Flea prevalence	Flea intensity	Mean Flea abundance
**South Dakota BTPD**							
** Buffalo Gap‐1**	BGSD‐1	792	467	246	52.68	3.22	1.70
** Buffalo Gap‐2**	BGSD‐2	682	397	228	57.43	2.99	1.72
** Lower Brule**	LBSD‐1	2594	487	404	82.96	6.42	5.33
** Wind Cave**	WCSD‐1	914	324	187	57.72	4.89	2.82
**Montana** BTPD							
** Charles M. Russell‐1**	CMR‐1	912	574	351	61.15	2.60	1.59
** Charles M. Russell‐2**	CMR‐2	700	462	264	57.14	2.65	1.52
** Charles M. Russell‐3**	CMR‐3	1736	618	493	79.77	3.52	2.81
** Charles M. Russell‐4**	CMR‐4	1270	628	443	70.54	2.87	2.02
** Charles M. Russell‐5**	CMR‐5	1666	344	235	68.31	7.09	4.84
**Texas** BTPD							
** Rita Blanca‐1**	RBTX‐1	1477	197	196	99.49	7.54	7.50
** Rita Blanca‐2**	RBTX‐2	1210	184	184	100.00	6.58	6.58
**Arizona** GPD							
**Espee Ranch**	ERAZ‐1	970	353	235	66.57	4.13	2.75
**Utah** UPD							
** Cedar City‐1**	CCUT‐1	152	144	61	42.36	2.49	1.06
** Cedar City‐2**	CCUT‐2	332	232	149	64.22	2.23	1.43
** Cedar City‐3**	CCUT‐3	300	280	147	52.50	2.04	1.07
** High elevation‐1**	HEUT‐1	636	291	188	64.60	3.38	2.19
** High elevation‐2**	HEUT‐2	872	369	238	64.50	3.66	2.36
** High elevation‐3**	HEUT‐3	1464	336	240	71.43	6.10	4.36
** High elevation‐4**	HEUT‐4	284	214	92	42.99	3.09	1.33
**Utah** WTPD							
** Coyote Basin‐1**	CBUT‐1	409	193	114	59.07	3.59	2.12
** Coyote Basin‐2**	CBUT‐2	423	236	114	48.31	3.71	1.79
**Wyoming** WTPD							
** Pitchfork Ranch‐1**	PRWY‐1	207	475	115	24.21	1.80	0.44
** Pitchfork Ranch‐2**	PRWY‐2	213	443	105	23.70	2.03	0.48

### 3.1 Relationship of flea abundance and presence/absence with covariates

**Figure 2:**
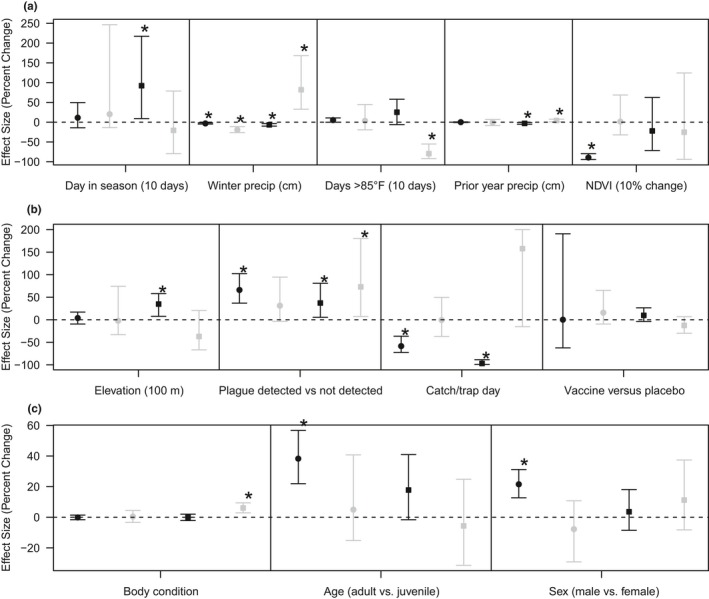
Effect sizes (represented as average percent change in flea abundance) as a function of (a) climate and environmental variables (precip=precipitation, see Table 1 for description of parameters), (b) nonplot‐level variables, and (c) characteristics of individual prairie dogs for black‐tailed prairie dog (*Cynomys ludovicianus*; black dots)*,* Gunnison's prairie dog (*Cynomys gunnisoni*; gray dots), Utah prairie dog (*Cynomys parvidens*; black squares), white‐tailed prairie dog (*Cynomys leucurus*; gray squares). *Indicates effect sizes greater than zero

**Figure 3:**
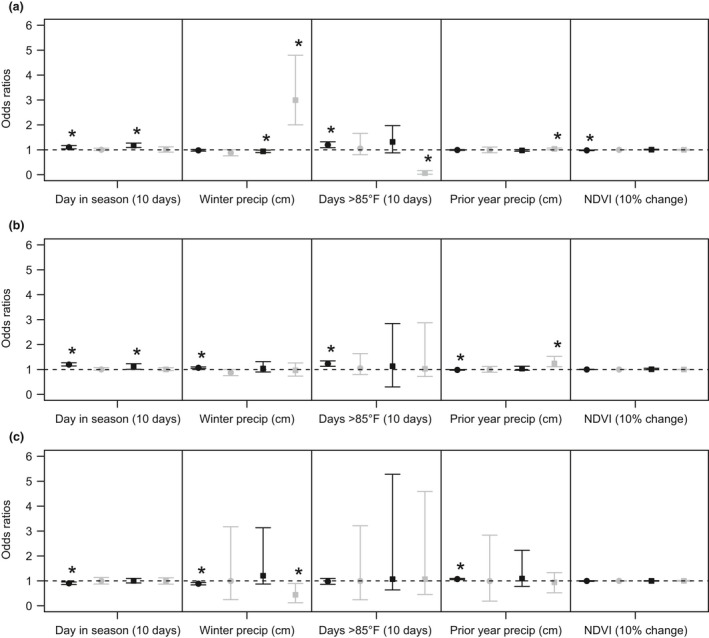
Odds ratios from results of logistic regression relating climate and weather variables (precip=precipitation, see Table 1 for description of parameters) to (a) flea presence (all flea species), (b) *Oropsylla hirsuta* presence*,* and c) *Pulex simulans* presence for black‐tailed prairie dog (*Cynomys ludovicianus*; black dots)*,* Gunnison's prairie dog (*Cynomys gunnisoni*; gray dots*),* Utah prairie dog (*Cynomys parvidens*; black squares), white‐tailed prairie dog (*Cynomys leucurus*; gray squares). *Indicates odds ratios that do not overlap one

**Figure 4:**
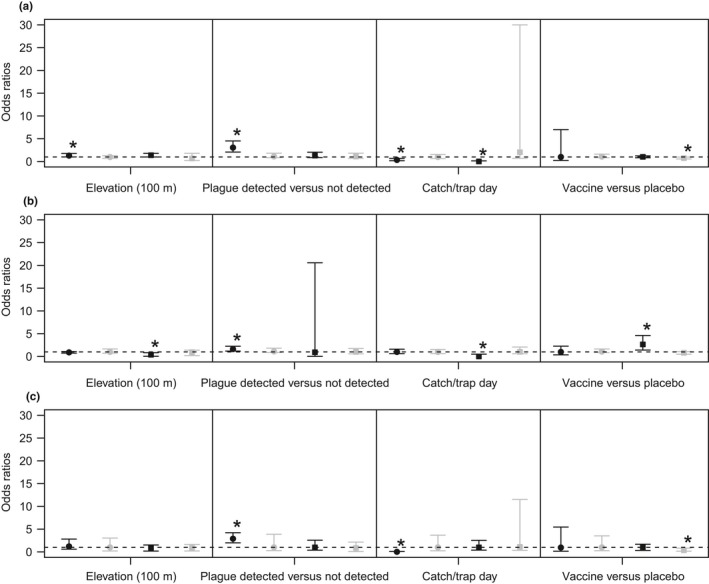
Odds ratios from results of logistic regression relating plot‐level variables (see Table 1 for description of parameters) to (a) flea presence (all flea species), (b) *Oropsylla hirsuta* presence, and (c) *Pulex simulans* presence for black‐tailed prairie dog (*Cynomys ludovicianus*; black dots)*,* Gunnison's prairie dog (*Cynomys gunnisoni*; gray dots)*,* Utah prairie dog (*Cynomys parvidens*; black squares), white‐tailed prairie dog (*Cynomys leucurus*; gray squares). * Indicates odds ratios that do not overlap one

**Figure 5:**
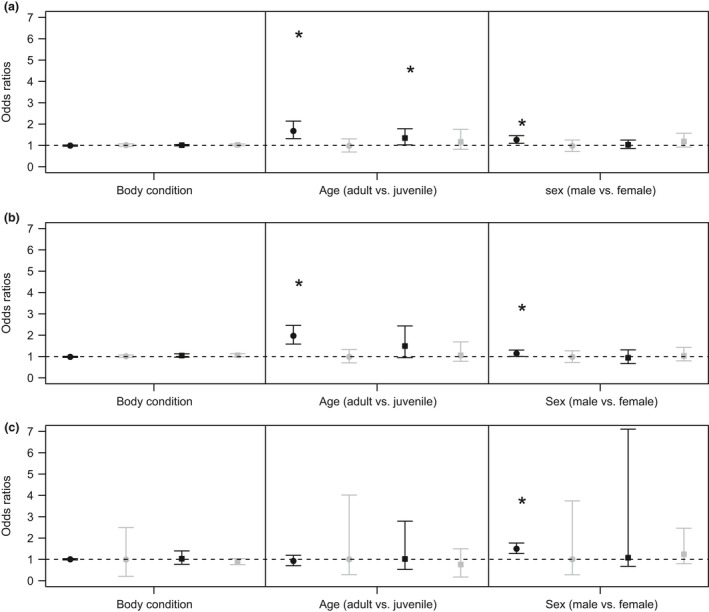
Odds ratios from results of logistic regression relating individual characteristics of prairie dogs (see Table 1 for description of parameters) to (a) total flea abundance, (b) *Oropsylla hirsuta* abundance, and (c) *Pulex simulans* abundance for black‐tailed prairie dog (*Cynomys ludovicianus*; black dots), Gunnison's prairie dog (*Cynomys gunnisoni*; gray dots), Utah prairie dog (*Cynomys parvidens*; black squares), white‐tailed prairie dog (*Cynomys leucurus*; gray squares). * Indicates odds ratios that do not overlap one

Results of our regression analyses on flea abundance and flea presence/absence for all fleas, *O. hirsuta*, and *P. simulans* indicate that multiple factors are associated with the abundance of fleas and that the relationship of some of these factors varies among prairie dog species (Figures 2, 3, 4, 5). Overall, area under the curve statistics for logistic regression models indicated an adequate fit for all fleas (0.72 BTPD, 0.61 GPD, 0.69 UPD, 0.77 WTPD), *O. hirsuta* (0.71 BTPD, 0.61 GPD, 0.94 UPD, 0.94 WTPD), and *P. simulans* (0.87 BTPD, 0.99 UPD, 0.97 WTPD; no *P. simulans* were detected on GPD). Posterior predictive checks on the parameters of the negative binomial distribution (mean and clustering parameter) also indicated adequate fits with p = 0.35–0.78 for comparisons of the observed mean and k for all prairie dog species to 1,000 simulated predictions from the model for total fleas (i.e., 35–78% percent of the time the values were above or below the true value; ideally 50% of the values are above and 50% below). For *O. hirsuta* and *P. simulans,* negative binomial regressions were only conducted for BTPD. Posterior predictive checks indicated adequate fits for these models as well with p‐values of 0.27 and 0.21 for the observed means and 0.61 and 0.88 for the observed clustering parameters for *O. hirsuta* and *P. simulans*, respectively.

### 3.2 Climate and weather variables

Multiple weather and climate‐related variables were associated with flea abundance and presence/absence. Total flea abundance increased 92% (95% Credible Interval [C.I.] 9–217%) on UPD pairs over every 10‐day period (Figure 2). The odds of a prairie dog having at least one flea increased by 1.10 (95% C.I. 1.03–1.17) for BTPD and 1.16 (95% C.I. 1.09–1.26) for UPD as days in the season increased (Figure 3). In addition, on BTPD pairs, *O. hirsuta* abundance increased an average of 214% (95% C.I. 88–392) with every 10 days later in the year (Table 3), while *P. simulans* abundance decreased 66% (95% C.I. 50–78%) (Table 3). On UPD pairs, day of sampling was associated with an increase in the odds of *O. hirsuta* presence (1.01, 95% C.I. 1.12–1.23) but not *P. simulans*.

**Table 3:** Effect sizes for results of negative binomial regressions estimating the associations between *Oropsylla hirsuta* and *Pulex simulans* abundance on BTPD. Effects are represented as a percent change. LCI: lower credible interval, UCI: upper credible interval

	*Oropsylla hirsuta*			*Pulex simulans*		
	Median	2.5% LCI	97.5% UCI	Median	2.5% LCI	97.5% UCI
Climate and weather variables						
Day in season (10 days)	214.36	88.17	392.51	−66.66	−77.98	−50.55
Winter precipitation (cm)	0.51	−1.59	2.66	−7.33	−10.4	−4.26
Number of days >85°F (10 days)	21.75	13.82	30.54	−1.99	−10.09	6.75
Prior year precipitation (cm)	−2.27	−3.18	−1.32	7.17	5.65	8.67
NDVI (10% change)	−25.66	−66.69	24.03	−55.11	−89.36	21.53
Pair‐level characteristics						
Elevation (100 m)	−11.09	−27.75	3.9	−5.37	−48.72	88.84
Plague detected vs no plague detected	61.65	25.47	109.15	73.49	35.78	121.21
Catch/trap days	−9.16	−42.65	30.09	−93.93	−97.09	−87.39
Vaccine vs placebo	4.51	−46.62	133.21	2.98	−80.3	1100.99
Individual characteristics						
Adult vs juvenile	55.4	32.32	83.46	−5.08	−20.39	12.48
Body condition (wt(g)/foot length (mm))	−0.04	−2.06	1.89	0.1	−1.89	2.15
Male vs female	12.98	2.6	24.41	45.72	30.98	62.17

With a 10% increase in NDVI, total flea abundance decreased by 89% (95% C.I. 80–95%) on BTPD pairs, but these differences were not significant for individual flea species (Figures 2 and 3). For every 10 days with temperatures over 85°F, total flea abundance decreased by 80% (95% C.I. 55–92%) and the odds of flea presence decreased by 0.06 (95% C.I. 0.02–0.16) for WTPD; however, these results were not evident for individual flea species (Figure 3). For the same change in number of degree days, total flea abundance increased by 5% (95% C.I. 0–11%) and the odds of flea presence increased by 1.19 (95% C.I. 1.08–1.31) for BTPD; these differences were driven largely by changes in *O. hirsuta* with average increases in abundance of 21% (95% C.I. 14–20%; Table 3) and increases in odds of *O. hirsuta* presence of 1.23 (95% C.I. 1.13–1.34) (Figure 3).

Precipitation amounts also had varying effects on flea abundance (Figures 2 and 3). For every 1 cm increase in winter precipitation on WTPD pairs, total flea abundance increased by 83% (95% C.I. 33–165%) with odds of flea presence increasing by 2.99 (95% C.I. 2.00–4.80). However, odds of *P. simulans* presence declined with increasing precipitation (0.44, 95% C. I. 0.12–0.89) on these plots. On BTPD plots, flea abundance declined by 3% (95% C.I. 2–5%) and odds of flea presence decreased by 0.98 (95% C.I. 0.95–1.00) with increasing winter precipitation. These results were largely driven by declines in *P. simulans* with an average decline of 7% (95% C.I. 4–10%) with increasing winter precipitation. On GPD and UPD pairs, every 1 cm increase in winter precipitation led to odds of flea presence decreasing by 0.88 (95% C.I. 0.76–1.00) and 0.94 (95% C.I. 0.89–0.99), respectively, with corresponding decreases in total flea abundance of 19% (95% C.I. 11–27%) on GPD plots and 7% (95% C.I. 4–10%) on UPD plots. Odds of *O. hirsuta* presence also declined with increasing winter precipitation on GPD plots (0.87, 95% C.I. 0.75–1.00), but not UPD plots. Prior year precipitation was related to increasing odds of *P. simulans* presence (1.07, 95% C.I. 1.05–1.10) on BTPD plots and increasing odds of O*. hirsuta* presence (1.24, 95% C.I. 1.11–1.52) on WTPD plots. *O. hirsuta* flea abundance declined by an average of 2% (95% C.I. 1–3%), while *P. simulans* abundance increased an average of 7% (95% C.I. 6–9%) for every 1 cm increase in prior year precipitation on BTPD plots (Table 3).

### 3.3 Plot‐level characteristics

Plot‐level variables also indicated associations with flea abundance and presence/absence. The presence of plague was positively associated with flea abundance for all species except GPD (Figure 2). In addition, *O. hirsuta* and *P. simulans* abundance was associated with plague detection for BTPD (Table 3). Odds of flea presence increased for every 100 m increase in elevation on BTPD (1.29, 95% C.I. 1.01–1.77) and UPD (1.39, 95% C.I. 1.00–1.79) plots (Figure 4), and flea abundance increased by an average of 35% (95% C.I. 8–58%) on UPD plots as well (Figure 2). Odds of *O. hirsuta* presence, however, declined on UPD plots (0.37, 95% C.I. 0.04–0.83) with elevation increases. For every 1 unit increase in catch per unit effort, odds of flea presence was 0.02 (95% C.I. 0.00–0.14) for UPD and 0.35 (95% C.I. 0.17–0.70) for BTPD plots, while average flea abundance declined 97% (95% C.I. 89–99%) and 58% (95% C.I. 37–72%) on UPD and BTPD plots, respectively. These results were driven by declines in *O. hirsuta* on UPD plots and *P. simulans* on BTPD plots (Figure 4, Table 3). At last, on average, the odds of flea presence was lower on vaccine versus placebo plots for WTPD (0.74, 95% C.I. 0.56–0.97) only, and these results were reflected in declines in the odds of *P. simulans* presence (0.36, 95% C.I. 0.16–0.83). Increases in odds of *O. hirsuta* presence were detected on UPD vaccine versus placebo plots (2.65, 95% C.I. 1.41–4.65); however, *O. hirsuta* were only found on CCUT sites and not HEUT. The CCUT sites were small, <4 hectares in size, therefore the effects of the vaccine may have been swamped by movement on and off the site.

### 3.4 Individual characteristics of prairie dogs

Flea abundance increased by 6% (95% C.I. 3–9%) for every 1 unit increase in body condition (g/mm) for WTPD, but no other species (Figure 2). Odds of flea presence increased by 1.69 (95% C.I. 1.33–2.14) and 1.34 (95% C.I. 1.03–1.75) for adult versus juvenile BTPD and UPD respectively (Figure 5), and adult BTPD on average had 38% (95% C.I. 21–57%) more fleas than juveniles. Odds of *O. hirsuta* presence also increased on adult versus juvenile BTPD by 1.97 (95% C.I. 1.58–2.46) (Figure 5), and average *O. hirsuta* abundance was 55% (95% C.I. 32–83%) higher on adults. Male BTPD on average had 21% (95% C.I. 13–31%) more total fleas, 13% (95% C.I. 3–24%) more *O. hirsuta*, and 43% (95% C.I. 31–62%) more *P. simulans* than females.

### 3.5 Flea species composition

**Figure 6:**
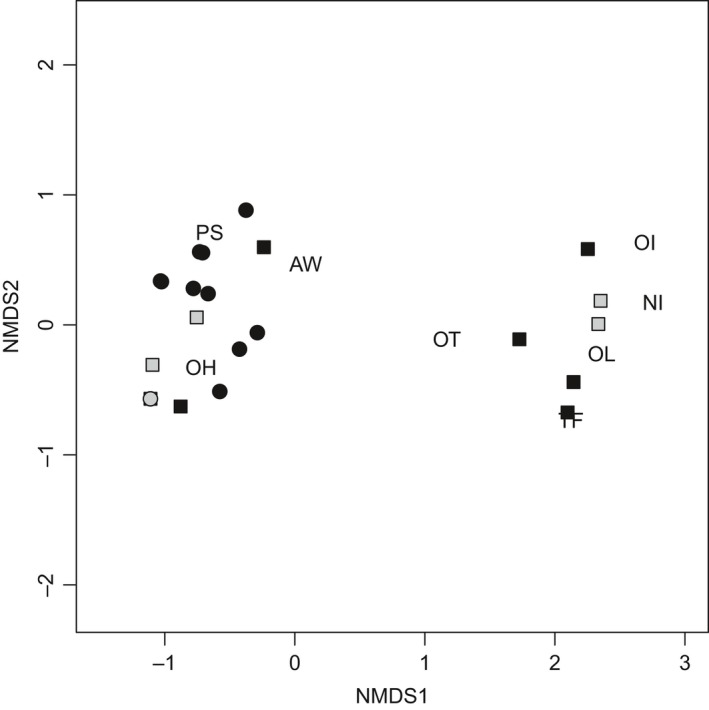
Nonmetric multidimensional scaling of flea communities, including black‐tailed prairie dog (*Cynomys ludovicianus*; black dots), Gunnison's prairie dog (*Cynomys gunnisoni*; gray dots), Utah prairie dog (*Cynomys parvidens*; black squares), white‐tailed prairie dog (*Cynomys leucurus*; gray squares). Flea species are indicated in large capital letters. OH: *Oropsylla hirsuta*; OT: *O. tuberculata*; OL: *O. labis*; OI: *O. idahoensis*; TF: *Thrassis francisi*; PS: *Pulex simulans*; AW: *Aetheca wagneri*; and NI: *Neopsylla inopina*

Results of our NMDS reflect results of our regression analyses (Figure 6). High elevation pairs in Wyoming and Utah (Appendix 7) had distinct flea communities compared with the rest of the study pairs. Communities dominated by *O. hirsuta* included lower elevation UPD pairs (CCUT) and WTPD pairs in Utah, BTPD pairs in South Dakota, and the one GPD pair in Arizona, while *P. simulans* was associated with BTPD pairs in Montana and Texas, as well as one WTPD pair in Utah. Stress values indicate adequate fit (0.98).

### 3.6 Plague detections

The incidence of prairie dogs with *Y. pestis* detections in fleas was low (*n* = 64 prairie dogs with positive fleas out of 5,024 prairie dogs sampled from 4,218 individual prairie dogs). Fleas positive for *Y. pestis* were found on BTPD plots in Montana (2016) and Texas (2014 and 2015), WTPD plots in Wyoming (2015 and 2016) and Utah (2015 and 2016), high elevation UPD plots (2014 and 2015), and the one GPD plot in Arizona (2014) (Appendix 4). Twenty‐five of the prairie dogs with *Y. pestis* positive flea pools were found on placebo plots and 39 on vaccine plots which represent less than 2% of all prairie dogs tested. *Y. pestis* positive fleas were found on juvenile and adult prairie dogs of both sexes and in flea pools of 7 different species including *P. simulans*,* O. hirsuta*,* O. tuberculata*,* O. labis*,* O. idahoensis*,* Thrassis francisi*, and *Neopsylla inopina* (*Y. pestis* was not detected in any *A. wagneri* pools). Thirty‐three plague‐positive carcasses were collected from seven placebo plots and five vaccine plots over the 4‐year period (Table 4, Appendix 4).

**Table 4:** Number of plague‐positive carcasses collected from study plots. Carcasses were considered to be plague positive if *Yersinia pestis* was cultured from tissues or detected by PCR analysis

Pair	Species	Treatment	2014	2015	2016
CBUT‐2	White‐tailed	Placebo		1	
CCUT‐2	Utah	Placebo			2
CMR‐1	Black‐tailed	Placebo			3
CMR‐2	Black‐tailed	Placebo		1	
HEUT‐2	Utah	Placebo	3	4	
HEUT‐3	Utah	Placebo		1	
PRWY‐1	White‐tailed	Placebo			1
CBUT‐2	White‐tailed	Vaccine		4	
ERAZ‐1	Gunnison's	Vaccine	2		
HEUT‐2	Utah	Vaccine	5	2	
PRWY‐1	White‐tailed	Vaccine			1
PRWY‐2	White‐tailed	Vaccine			3

Flea abundance was higher on animals with PCR‐positive flea pools (mean = 11.7, SD = 14.1) than animals without PCR‐positive fleas (mean = 3.9, SD = 5.1). Plague detections in *P. simulans* only occurred at plots where plague was also detected in *O. hirsuta*. Plague‐positive fleas (*T. francisi*,* O. labis*,* O. idahoensis*, and *O. tuberculata*) were found on PRWY and HEUT plots, but no *O. hirsuta* were detected on these plots. Overall, plague was detected in carcasses or fleas at 18 (9 placebo plots and 9 vaccine plots, not all paired) of 46 plots (see Rocke et al., 2017 for more details). Logistic regression estimates indicated that no covariates were significantly associated with initial plague outbreaks on placebo plots.

## 4 DISCUSSION

Many factors were associated with flea abundance on prairie dogs, indicating the complexity of host‐vector‐disease dynamics in this system. Our results indicate that local weather factors influenced flea abundance, potentially by providing environmental conditions suitable for flea reproduction. As expected, however, environmental factors had different effects at different locations due to the wide range of environmental conditions and geographic locations of our study pairs. Differences in the direction of the relationship of environmental covariates with flea abundance for different prairie dog species is likely the result of differences in average local conditions and differences in the biology of the prairie dog and flea species in question. Abundance of fleas is likely more important for epizootic plague, versus the presence or absence of fleas on hosts at a location. High flea loads have been shown to be important for epizootic transmission via blocked fleas (Lorange et al., 2005). Abundant fleas are also likely to result in more efficient early‐phase transmission (Eisen et al., 2006), whereas flea presence merely indicates that at least one flea is present on the host. Therefore, a large number of hosts with low flea abundance (i.e., high flea prevalence) may not result in plague outbreaks. We did not find any relationship between local weather conditions and the likelihood of plague outbreaks. However, our sample sizes were small and geographically varied. It is likely that conditions under which plague epizootics occur are as locally‐specific as the conditions that result in high flea abundance.

Winter precipitation increases resulted in decreases in flea abundance for all species except WTPD, while overall flea abundance decreased with increasing number of days with temperatures above 85°F on WTPD plots but increased with warmer temperatures on BTPD plots. These results may reflect differences in species composition or local weather conditions. Salkeld and Stapp (2008) found increasing *O. hirsuta* and decreasing *O. tuberculata* populations in prairie dog burrows with increasing temperature, which may explain some of these differences. Soil type is an additional factor that may influence flea abundance. Savage et al. (2011) found that soils with increased soil moisture holding capacity were related to the incidence of plague epizootics; however, Salkeld and Stapp (2008) found no relationship between *O. hirsuta* abundance and soil type. Despite the overall increase in flea abundance on WTPD pairs, *P. simulans* abundance decreased with increasing winter precipitation on these plots (as well as BTPD plots), indicating that the increases in flea abundance on WTPD plots with winter precipitation are driven by other flea species. In addition, it is likely that flea abundance is optimal in a mid‐range of conditions. On WTPD plots, average winter precipitation was roughly 2 cm greater than on BTPD plots, indicating that greater precipitation on these wet plots led to decreases in flea abundance in contrast to plots that received lesser amounts of precipitation.

Several possible mechanisms may explain the effects of local weather on flea abundance and plague outbreaks. Local weather factors may affect flea abundance directly by influencing flea mortality or reproductive rates (Krasnov, Khokholova, Fielden, & Burdelova, 2001) or indirectly by changing forage availability and affecting host body condition and/or grooming behavior (Eads et al., 2016). Flea abundance on BTPD plots declined with increasing NDVI, supporting the hypothesis that better forage conditions on BTPD plots may lead to lower flea abundance. In our data set, body condition was not strongly associated with flea abundance of most species, except WTPD, and for this species, animals with better body condition tended to have more fleas, possibly due to their larger size. It is possible that body condition may increase in importance during drought conditions which were not observed on our plots during 2013–2016. One study noted that BTPD that survived plague outbreaks tended to have better body condition than prior to the die‐off, potentially due to less competition for forage (Pauli, Buskirk, Williams, & Edwards, 2006). This observation may explain the relationship we observed between flea abundance on plague‐positive plots and body condition in our study. We did not have direct before‐after comparisons; however, there was no difference in average body condition between animals captured on plots with plague detections (mean body condition = 14.0, SD = 4.4), compared to plots with no plague detection (mean body condition = 14.5, SD = 4.6).

Previous research has indicated that trends in flea abundance during a season are highly dependent on the flea community (Tripp et al., 2009). Different flea species have different seasonal peaks in abundance, with *O. tuberculata* tending to be more common in early spring, *O. idahoensis* in midsummer (Anderson & Williams, 1997), and *O*. *hirsuta* later in the summer (Tripp et al., 2009). Our study pairs were sampled between June and November over a short period of time, thus flea peaks in early spring were missed. In addition, different pairs were sampled at different times of the year (Appendix 1), with pairs in Wyoming sampled the earliest on average and pairs in Texas surveyed the latest, an average of 125 days later. Therefore, our result that flea abundance increased over time may be reflective of seasonal differences in the timing of sampling between pairs and/or the location of the pair along the latitudinal gradient. Our data indicated that total flea abundance (specifically *O. hirsuta*) tended to increase during summer months for UPD and BTPD, while abundance of *P. simulans* declined on BTPD plots. Most of our BTPD colonies were located at the more northern range of our study pairs; therefore, optimal conditions for flea growth may have occurred later in the season at these pairs.

Flea community composition and diversity also varied by location. The highest elevation pairs (2 WTPD pairs in Wyoming, PRWY, and 4 UPD pairs in Utah, HEUT), with elevation >2000 m, (Appendix 7) had the highest diversity of flea species, although they were the only pairs where *O. hirsuta* was not found. Instead, two other *Oropsylla* species, *O. tuberculata* and *O. labis,* were commonly found, while *O. idahoensis* was commonly found on both Wyoming pairs but only the highest elevation pair in Utah (HEUT‐4). In addition, HEUT pairs were the only ones where *T. francisi* was commonly found. These results may indicate that at high elevation, different flea species associated with different small mammal communities play a role in the maintenance of plague in the system.

On UPD and BTPD plots, increasing catch per unit effort was associated with decreasing flea abundance, potentially indicating that larger prairie dog populations resulting from improved forage conditions lead to lower flea abundance. However, this relationship may also be driven by plague dynamics. Higher flea abundance on plague‐affected plots (which also have lower catch per unit effort) may be the result of fleas congregating on surviving animals (Tripp et al., 2009). It is also possible that the combination of high flea abundance and high prairie dog abundance leads directly to plague epizootics and is therefore rarely observed (i.e., once these conditions are met there is a plague outbreak which drives prairie dog densities lower). Lastly, the combination of high densities of animals in summers that are drier than average could lead to poorer condition of animals overall, leading to higher susceptibility to plague the following summer, particularly if forage availability (as indicated by NDVI) is low.

Changing climate conditions will likely affect aspects of both flea and host communities, including population densities and species composition, and lead to changes in plague dynamics. We expected that plague outbreaks would be less likely when temperatures were high (Snäll et al., 2008); however, we did not observe this relationship in our data set. High temperatures have been observed to interfere with transmission of *Y. pestis* in rat fleas (Cavanaugh, 1971) and may lead to decreases in plague outbreaks with warming temperatures. In an alternative manner, hosts and plague vectors may expand their range northward, thus shifting the range of *Y. pestis* outbreaks (Nakazawa et al., 2007). A meta‐analysis recently provided evidence for the effects of local factors on disease outbreaks, including the specific composition of the host and vector community (Salkeld, Padgett, & Jones, 2013). Our results support the hypothesis that local conditions, including host, vector, and environmental factors, influence the likelihood of plague outbreaks, and that predicting changes to plague dynamics under climate change scenarios will require consideration of both host and vector responses to local factors.

## CONFLICT OF INTEREST

None declared.

## AUTHOR CONTRIBUTIONS

T.R., D.T., and R.R. conceived the ideas and design of the study; R.R. and T.R. designed the methodology; R.A. and D.T. collected the data; R.R. analyzed the data and R.R., T.R., D.T., and R.A. contributed to analysis interpretations; R.R. led the writing of the manuscript. All authors contributed critically to the drafts and gave final approval for publication.

## DATA ACCESSIBILITY

Data are available at https://doi.org/10.5066/f7tm79ck.

## APPENDIX 1

### Sampling times for study sites. BTPD is black‐tailed prairie dog (*Cynomys ludovicianus*)*,* WTPD is white‐tailed prairie dog (*Cynomys leucurus*), UPD is Utah prairie dog (*Cynomys parvidens*), and GPD is Gunnison's prairie dog (*Cynomys gunnisoni*). NS: not sampled

**Table:**

Study area and species	2013		2014	2015	2016	
	Prebait	Postbait	Postbait	Postbait	Prebait	Postbait
**South Dakota** BTPD						
** Buffalo Gap‐1**	June	July	July	July	NS	NS
** Buffalo Gap‐2**	June	July	July	July	NS	NS
** Lower Brule**	July	August	August	August	NS	August
** Wind Cave**	June	July	July	July	NS	NS
**Montana** BTPD						
** Charles M. Russell‐1**	June	July	July	July	June	July
** Charles M. Russell‐2**	July	July	July	July	June	July
** Charles M. Russell‐3**	NS	August	July	July	June	August
** Charles M. Russell‐4**	June	July	July	July	June	August
** Charles M. Russell‐5**	July	August	August	August	NS	NS
**Texas** BTPD						
** Rita Blanca‐1**	August	October	October	October	NS	NS
** Rita Blanca‐2**	August	November	October	October	NS	NS
**Arizona** GPD						
** Espee Ranch**	June	July	July; August	July	NS	July
**Utah** UPD						
** Cedar City‐1**	June	July‐August	September	NS	NS	NS
** Cedar City‐2**	July	August	July	July	June	NS
** Cedar City‐3**	July	August	September	August	NS	September
** High Elevation‐1**	NS	June	July‐August	July	July	NS
** High Elevation‐2**	NS	June‐July	July‐August	July	July	NS
** High Elevation‐3**	NS	July	July‐August	July	July‐August	NS
** High Elevation‐4**	NS	July‐August	August	August	August	NS
**Utah** WTPD						
** Coyote Basin‐1**	June	July	July	July; August	NS	July
** Coyote Basin‐2**	June	July‐August	July	July	NS	July
**Wyoming** WTPD						
** Pitchfork Ranch‐1**	June	July	June	June	NS	June
** Pitchfork Ranch‐2**	June	July	July	July	NS	July

## APPENDIX 2

### Number of trap days. BTPD is black‐tailed prairie dog (*Cynomys ludovicianus*)*,* WTPD is white‐tailed prairie dog (*Cynomys leucurus*), UPD is Utah prairie dog (*Cynomys parvidens*), and GPD is Gunnison's prairie dog (*Cynomys gunnisoni*). NS: not sampled

**Table:**

Plot	Treatment	2013	2014	2015	2016
**South Dakota** BTPD					
** Buffalo Gap‐1**	Placebo	330	237	339	NS
Vaccine	255	214	210	NS
** Buffalo Gap‐2**	Placebo	330	248	190	NS
Vaccine	240	234	230	NS
** Lower Brule‐1**	Placebo	1,446	999	868	1,200
Vaccine	1,513	968	972	1,200
** Wind Cave‐1**	Placebo	353	390	379	NS
Vaccine	315	363	252	NS
**Montana** BTPD					
** Charles M. Russell‐1**	Placebo	332	194	176	960
Vaccine	342	206	190	960
** Charles M. Russell‐2**	Placebo	228	222	190	960
Vaccine	212	197	184	961
** Charles M. Russell‐3**	Placebo	216	226	201	1,200
Vaccine	220	202	199	1,200
** Charles M. Russell‐4**	Placebo	217	213	204	1,200
Vaccine	213	205	176	1,200
** Charles M. Russell‐5**	Placebo	188	191	149	NS
Vaccine	190	201	146	NS
**Texas** BTPD					
** Rita Blanca‐1**	Placebo	119	89	90	NS
Vaccine	117	89	89	NS
** Rita Blanca‐2**	Placebo	120	90	90	NS
Vaccine	120	90	90	NS
**Arizona** GPD					
** Espee Ranch**	Placebo	399	627	434	450
Vaccine	402	642	449	531
**Utah** UPD					
** Cedar City‐1**	Placebo	222	225	NS	NS
Vaccine	225	228	NS	NS
** Cedar City‐2**	Placebo	225	225	225	4
Vaccine	219	225	225	1
** Cedar City‐3**	Placebo	189	225	225	225
Vaccine	225	225	225	225
** High Elevation‐1**	Placebo	504	623	498	336
Vaccine	477	619	484	390
** High Elevation‐2**	Placebo	560	712	347	420
Vaccine	560	730	360	420
** High Elevation‐3**	Placebo	375	506	281	480
Vaccine	350	449	298	480
** High Elevation‐4**	Placebo	504	264	286	192
Vaccine	630	242	268	384
**Utah** WTPD					
** Coyote Basin‐1**	Placebo	396	297	487	150
Vaccine	388	272	351	150
** Coyote Basin‐2**	Placebo	520	287	176	120
Vaccine	270	387	551	180
**Wyoming** WTPD					
** Pitchfork Ranch‐1**	Placebo	762	1,249	1,216	960
Vaccine	742	1,264	1,153	1,280
** Pitchfork Ranch‐2**	Placebo	1,553	1,262	1,186	1,280
Vaccine	1,486	1,261	1,184	960

## APPENDIX 4

### Number of prairie dogs sampled (number of unique animals if different from number sampled), number of prairie dogs with plague‐positive flea pools (indicated by +), number of plague‐positive carcasses (boldface). BTPD is black‐tailed prairie dog (*Cynomys ludovicianus*)*,* WTPD is white‐tailed prairie dog (*Cynomys leucurus*), UPD is Utah prairie dog (*Cynomys parvidens*), and GPD is Gunnison's prairie dog (*Cynomys gunnisoni*). NS: not sampled

**Table:**

Study area and species	2013	2014	2015	2016
**South Dakota** BTPD				
** Buffalo Gap‐1**	142 (130)	210	115	NS
** Buffalo Gap‐2**	124 (121)	169	104	NS
** Lower Brule**	175 (152)	106	100	106 (105)
** Wind Cave**	122 (106)	100	102 (100)	NS
**Montana** BTPD				
** Charles M. Russell‐1**	102 (97)	106	99	267 (227) 5+ **1**
** Charles M. Russell‐2**	111 (101)	113	124	114 (87) 11+ **1**
** Charles M. Russell‐3**	101	128	127	262 (203)
** Charles M. Russell‐4**	131 (119)	108	109	280 (236)
** Charles M. Russell‐5**	119 (106)	112	113	NS
**Texas** BTPD				
** Rita Blanca‐1**	120 (113)	58 1+	19 10+	NS
** Rita Blanca‐2**	93 (82)	38	53	NS
**Arizona** GPD				
** Espee Ranch**	107 (100)	77 (50) 6+ **2**	70 (68)	96 (92)
**Utah** UPD				
** Cedar City‐1**	86 (77)	58	NS	NS
** Cedar City‐2**	89 (79)	68	70	NS
** Cedar City‐3**	106 (90)	52	94	28
** High Elevation‐1**	113	92 (87)	39	47 (42)
** High Elevation‐2**	123	182 (181) 2+ **8**	34 4+ **4**	30
** High Elevation‐3**	108	96 (95)	84 8+	48
** High Elevation‐4**	62	37	95	20
**Utah** WTPD				
** Coyote Basin‐1**	56 (53)	108 (107)	28 3+ **2**	1 1+
** Coyote Basin‐2**	73 (70)	95 (94)	65 6+	3
**Wyoming WTPD**				
** Pitchfork Ranch‐1**	151 (141)	132	119 2+	73 3+ **2**
** Pitchfork Ranch‐2**	140 (131)	121 (118)	121	61 2+ **3**

## APPENDIX 5

### Summary of mean flea abundance per prairie dog by flea species and location of prairie dog colony. Total number of fleas is in parentheses; if no total is listed, no fleas of that species were found. BTPD is black‐tailed prairie dog (*Cynomys ludovicianus*)*,* WTPD is white‐tailed prairie dog (*Cynomys leucurus*), UPD is Utah prairie dog (*Cynomys parvidens*), and GPD is Gunnison's prairie dog (*Cynomys gunnisoni*). OH: *Oropsylla hirsuta*; OT: *O. tuberculata*; OL: *O. labis*; OI: *O. idahoensis*; TF: *Thrassis francisi*; PS: *Pulex simulans*; AW: *Aetheca wagneri*; NI: *Neopsylla inopina*. Sampling periods and sample sizes are listed in Appendices S1 and S4, respectively

**Table:**

Study area and species	Year	OH	OT	OL	OI	TF	PS	AW	NI
**South Dakota** BTPD									
**Buffalo Gap‐1**	2013	3.15 (448)	0.09 (13)	0.00	0.00	0.00	0.00	0.01 (1)	0.00
	2014	0.75 (158)	0.00 (1)	0.00	0.00	0.00	0.00	0.00	0.00
	2015	1.48 (170)	0.00	0.00	0.00	0.00	0.00	0.00	0.00
**Buffalo Gap‐2**	2013	1.56 (194)	0.04 (5)	0.00	0.00	0.00	0.00	0.00	0.00
	2014	1.83 (309)	0.00	0.00	0.00	0.00	0.00	0.00	0.00
	2015	1.67 (174)	0.00	0.00	0.00	0.00	0.00	0.00	0.00
**Lower Brule**	2013	7.49 (1310)	0.00	0.00	0.00	0.00	0.00	0.00	0.00
	2014	3.63 (385)	0.00	0.00	0.00	0.00	0.00	0.00	0.00
	2015	6.63 (663)	0.00	0.00	0.00	0.00	0.00	0.00	0.00
	2016	2.21 (234)	0.00	0.00	0.00	0.00	0.00	0.00	0.00
**Wind Cave**	2013	4.54 (554)	0.00	0.00	0.00	0.00	0.00	0.00	0.00
	2014	0.89 (89)	0.00	0.00	0.00	0.00	0.00	0.00	0.00
	2015	2.61 (266)	0.01 (1)	0.00	0.00	0.00	0.00	0.01 (1)	0.00
**Montana** BTPD									
**Charles M. Russell‐1**	2013	0.69 (70)	0.08 (8)	0.00	0.00	0.00	1.39 (142)	0.00	0.00
	2014	0.31 (33)	0.01 (1)	0.00	0.00	0.00	0.09 (10)	0.00	0.00
	2015	1.02 (101)	0.03 (3)	0.00	0.00	0.00	0.34 (34)	0.00	0.00
	2016	1.17 (312)	0.01 (2)	0.00	0.00	0.00	0.73 (196)	0.00	0.00
**Charles M. Russell‐2**	2013	0.62 (69)	0.00	0.00	0.00	0.00	0.49 (54)	0.00	0.00
	2014	0.37 (42)	0.00	0.00	0.00	0.00	0.87 (98)	0.00	0.00
	2015	0.61 (76)	0.00	0.00	0.00	0.00	0.20 (25)	0.00	0.00
	2016	1.69 (193)	0.01 (1)	0.00	0.00	0.00	1.25 (142)	0.00	0.00
**Charles M. Russell‐3**	2013	1.96 (198)	0.00	0.00	0.00	0.00	1.46 (147)	0.00	0.00
	2014	0.45 (58)	0.00	0.00	0.00	0.00	3.59 (460)	0.02 (2)	0.00
	2015	1.95 (248)	0.00	0.00	0.00	0.00	1.28 (162)	0.00	0.00
	2016	0.92 (240)	0.01 (3)	0.00	0.00	0.00	0.83 (218)	0.00	0.00
**Charles M. Russell‐4**	2013	0.65 (85)	0.01 (1)	0.00	0.00	0.00	0.99 (130)	0.00	0.00
	2014	0.62 (67)	0.00	0.01 (1)	0.00	0.00	2.94 (318)	0.00	0.00
	2015	1.34 (146)	0.00	0.00	0.00	0.00	1.47 (160)	0.00	0.00
	2016	0.95 (266)	0.00 (1)	0.00	0.00	0.00	0.33 (91)	0.00	0.00
**Charles M. Russell‐5**	2013	0.30 (36)	0.01 (1)	0.00	0.00	0.00	0.62 (74)	0.00	0.00
	2014	2.76 (309)	0.01 (1)	0.01 (1)	0.00	0.00	4.47 (501)	0.00	0.00
	2015	3.64 (411)	0.02 (2)	0.00	0.00	0.00	2.92 (330)	0.00	0.00
**Texas** BTPD									
**Rita Blanca‐1**	2013	5.10 (612)	0.00	0.00	0.00	0.00	2.44 (293)	0.00	0.00
	2014	2.67 (155)	0.00	0.00	0.00	0.00	3.90 (226)	0.00	0.00
	2015	1.95 (37)	0.00	0.00	0.00	0.00	8.11 (154)	0.00	0.00
**Rita Blanca‐2**	2013	3.83 (356)	0.00	0.00	0.00	0.00	3.27 (304)	0.00	0.00
	2014	3.58 (136)	0.00	0.00	0.00	0.00	2.26 (86)	0.00	0.00
	2015	2.36 (125)	0.00	0.00	0.00	0.00	3.83 (203)	0.00	0.00
**Arizona** GPD									
**Espee Ranch**	2013	1.43 (153)	0.00	0.00	0.00	0.00	0.00	0.00	0.00
	2014	6.14 (473)	0.00	0.00	0.00	0.00	0.00	0.00	0.00
	2015	1.24 (87)	0.00	0.00	0.00	0.00	0.00	0.00	0.00
	2016	2.56 (253)	0.00	0.00	0.00	0.00	0.00	0.00	0.00
**Utah** UPD									
**Cedar City‐1**	2013	1.29 (111)	0.00	0.00	0.00	0.01 (1)	0.00	0.00	0.00
	2014	0.69 (40)	0.00	0.00	0.00	0.00	0.00	0.00	0.00
**Cedar City‐2**	2013	1.82 (162)	0.00	0.00	0.00	0.00	0.00	0.00	0.00
	2014	1.37 (93)	0.00	0.00	0.00	0.00	0.00	0.00	0.00
	2015	1.00 (70)	0.00	0.00	0.00	0.00	0.00	0.00	0.00
	2016	1.40 (7)	0.00	0.00	0.00	0.00	0.00	0.00	0.00
**Cedar City‐3**	2013	0.94 (100)	0.00	0.00	0.00	0.00	0.00	0.00	0.00
	2014	1.52 (79)	0.00	0.00	0.00	0.00	0.00	0.00	0.00
	2015	0.70 (66)	0.00	0.00	0.00	0.00	0.03 (3)	0.00	0.00
	2016	1.71 (48)	0.00	0.00	0.00	0.00	0.00	0.04 (1)	0.00
**High elevation‐1**	2013	0.00	0.05 (6)	0.08 (9)	0.00	0.85 (96)	0.00	0.00	0.00
	2014	0.00	0.08 (7)	0.41 (38)	0.00	2.86 (263)	0.00	0.00	0.00
	2015	0.00	0.00	0.49 (19)	0.00	2.69 (105)	0.00	0.00	0.00
	2016	0.00	0.30 (14)	0.32 (15)	0.00	1.09 (51)	0.00	0.00	0.00
**High elevation‐2**	2013	0.00	0.64 (79)	0.03 (4)	0.00	0.65 (80)	0.00	0.00	0.00
	2014	0.00	0.43 (78)	0.08 (15)	0.01 (1)	1.04 (189)	0.00	0.01 (1)	0.00
	2015	0.00	4.12 (140)	1.38 (47)	0.00	1.53 (52)	0.00	0.00	0.00
	2016	0.00	3.97 (119)	0.17 (5)	0.00	0.63 (19)	0.00	0.00	0.00
**High elevation‐3**	2013	0.00	0.34 (37)	0.08 (9)	0.00	1.13 (122)	0.00	0.00	0.00
	2014	0.00	0.30 (29)	0.34 (33)	0.01 (1)	4.42 (424)	0.00	0.00	0.00
	2015	0.00	0.44 (37)	0.26 (22)	0.00	3.45 (290)	0.00	0.00	0.00
	2016	0.00	1.75 (84)	0.29 (14)	0.02 (1)	5.77 (277)	0.00	0.00	0.00
**High elevation‐4**	2013	0.00	0.03 (2)	0.02 (1)	0.27 (17)	0.05 (3)	0.00	0.00	0.00
	2014	0.00	0.00	0.11 (4)	3.84 (142)	0.00	0.00	0.00	0.00
	2015	0.00	0.00	0.00	0.73 (69)	0.00	0.00	0.00	0.00
	2016	0.00	0.35 (7)	0.05 (1)	1.00 (20)	0.35 (7)	0.00	0.00	0.00
**Utah** WTPD									
**Coyote Basin‐1**	2013	0.36 (20)	0.04 (2)	0.00	0.00	0.00	0.05 (3)	0.00	0.00
	2014	0.83 (90)	0.00	0.00	0.00	0.00	0.53 (57)	0.00	0.00
	2015	4.86 (136)	0.00	0.00	0.00	0.00	1.00 (28)	0.00	0.00
	2016	70.00 (70)	0.00	0.00	0.00	0.00	0.00	0.00	0.00
**Coyote Basin‐2**	2013	0.36 (26)	0.00	0.00	0.00	0.00	0.01 (1)	0.00	0.00
	2014	0.63 (60)	0.00	0.00	0.00	0.00	0.00	0.00	0.00
	2015	4.92 (320)	0.00	0.00	0.00	0.00	0.00	0.00	0.00
	2016	5.33 (16)	0.00	0.00	0.00	0.00	0.00	0.00	0.00
**Wyoming** WTPD									
**Pitchfork Ranch‐1**	2013	0.00	0.00	0.05 (7)	0.05 (8)	0.00	0.00	0.00	0.03 (4)
	2014	0.00	0.04 (5)	0.01 (1)	0.08 (10)	0.00	0.00	0.00	0.00
	2015	0.00	0.06 (7)	0.15 (18)	0.33 (39)	0.00	0.00	0.00	0.06 (7)
	2016	0.00	0.41 (30)	0.47 (34)	0.42 (31)	0.00	0.00	0.00	0.07 (5)
**Pitchfork Ranch‐2**	2013	0.00	0.01 (1)	0.04 (6)	0.21 (29)	0.00	0.00	0.00	0.01 (1)
	2014	0.00	0.00	0.01 (1)	0.05 (6)	0.00	0.00	0.00	0.00
	2015	0.00	0.02 (3)	0.26 (31)	0.50 (60)	0.00	0.00	0.00	0.01 (1)
	2016	0.00	0.08 (5)	0.28 (17)	0.72 (44)	0.00	0.00	0.00	0.10 (6)

## APPENDIX 6

### Uncommon flea species identified on prairie dogs. BTPD is black‐tailed prairie dog (*Cynomys ludovicianus*)*,* WTPD is white‐tailed prairie dog (*C. leucurus*), UPD is Utah prairie dog (*C. parvidens*), and GPD is Gunnison's prairie dog (*C. gunnisoni*). See Hubbard, 1947 for references to typical hosts

**Table:**

Flea species	Typical host	Number of fleas	Site	Prairie dog species hosting flea
*Catallagia* sp.	Mouse	1	HEUT	UPD
*Eumolpianus eumolpi*	Chipmunk	7	HEUT	UPD
*Foxella ignota*	Pocket gopher	1	CCUT	UPD
*Hoplopsyllus affinis*	Rabbit	3	ERAZ	GPD
*Hoplopsyllus anomalus*	Ground squirrel	6	CCUT; HEUT; CBUT	UPD, WTPD
*Hystrichopsylla dippiei*	Mouse	1	PRWY	WTPD
*Oropsylla bruneri*	Ground squirrel	3	CMR	BTPD
*Oropsylla montana*	Ground squirrel	1	BGSD	BTPD
*Rhadinopsylla sectilis goodi*	Mouse, ground squirrel	15	HEUT; PRWY	UPD, WTPD
*Thrassis stanfordi*	Marmot, ground squirrel	7	HEUT	UPD

## APPENDIX 7

### Summary statistics of environmental factors associated with on‐host flea abundance on prairie dogs. Mean values of covariates by area. Standard deviations are in parentheses. BTPD is black‐tailed prairie dog (*Cynomys ludovicianus*)*,* WTPD is white‐tailed prairie dog (*C. leucurus*), UPD is Utah prairie dog (*C. parvidens*), and GPD is Gunnison's prairie dog (*C. gunnisoni*). NDVI: Normalized difference vegetation index (see Table 1 for explanation).

**Table:**

Study area and species	Flea load	NDVI	Catch per unit effort (animal/100 trap days)	Winter precipitation (cm)	Prior growing season precipitation (cm)	Days over 85°F	Elevation (m)
**South Dakota** BTPD							
**Buffalo Gap‐1**	1.70 (3.12)	5317.54 (584.82)	31.63 (16.72)	6.27 (4.15)	28.49 (2.73)	42.00 (6.99)	827
**Buffalo Gap‐2**	1.72 (2.96)	5107.97 (956.53)	30.07 (12.78)	6.27 (4.15)	28.49 (2.73)	42.00 (6.99)	822
**Lower Brule**	5.33 (8.06)	4406.52 (471.8)	13.3 (5.89)	8.22 (4.64)	33.06 (7.43)	45.00 (11.74)	545
**Wind Cave**	2.82 (6.05)	5514.27 (582.37)	19.79 (4.56)	9.6 (1.92)	37.35 (7.8)	30.33 (6.28)	1150
**Montana** BTPD							
**Charles M. Russell‐1**	1.59 (2.19)	4083.27 (488.76)	22.6 (11.00)	5.23 (2.1)	26.57 (7.82)	43.25 (8.15)	832
**Charles M. Russell‐2**	1.52 (2.3)	4038.79 (814.46)	32.33 (22.16)	5.23 (2.1)	26.57 (7.82)	43.25 (8.15)	860
**Charles M. Russell‐3**	2.81 (3.4)	2627.61 (494.11)	35.48 (13.54)	5.23 (2.1)	26.57 (7.82)	43.25 (8.15)	725
**Charles M. Russell‐4**	2.02 (2.6)	4442.67 (623.66)	31.18 (9.39)	5.23 (2.1)	26.57 (7.82)	43.25 (8.15)	720
**Charles M. Russell‐5**	4.84 (7.19)	3252.76 (782.12)	43.28 (15.36)	4.19 (1.00)	29.70 (6.21)	45.67 (8.07)	706
**Texas** BTPD							
**Rita Blanca‐1**	7.50 (5.9)	2763.77 (225.61)	29.05 (14.77)	2.38 (1.23)	19.30 (5.86)	80.67 (9.48)	1325
**Rita Blanca‐2**	6.58 (3.46)	2954.04 (566.05)	28.56 (6.82)	7.82 (3.59)	28.10 (5.19)	105.00 (4.1)	1220
**Arizona** GPD							
**Espee Ranch**	2.75 (4.38)	1975.38 (397.05)	10.34 (5.34)	6.01 (2.33)	16.45 (2.58)	50.75 (3.81)	1693
**Utah** UPD							
**Cedar City‐1**	1.06 (2.1)	3672.56 (761.01)	13.99 (5.54)	7.34 (2.43)	18.78 (3.09)	70.00 (1.15)	1741
**Cedar City‐2**	1.43 (1.65)	2440.6 (380.76)	33.16 (35.78)	8.59 (2.1)	15.51 (4.53)	71.25 (1.91)	1805
**Cedar City‐3**	1.07 (1.43)	3799.53 (898.5)	13.24 (9.33)	6.81 (3.21)	15.11 (2.54)	40.00 (6.97)	1940
**High elevation‐1**	2.19 (6.34)	2220.44 (472.26)	7.48 (3.58)	11.07 (3.59)	19.66 (5.52)	0 (0)	2575
**High elevation‐2**	2.36 (3.61)	3259.92 (747.25)	8.52 (4.71)	18.57 (1.99)	28.91 (3.04)	0 (0)	2797
**High elevation‐3**	4.36 (6.84)	3054.9 (412.94)	11.57 (7.69)	18.57 (1.99)	28.91 (3.04)	0 (0)	2772
**High elevation‐4**	1.33 (2.89)	4045.04 (685.55)	8.94 (6.00)	18.57 (1.99)	28.91 (3.04)	0 (0)	2864
**Utah** WTPD							
**Coyote Basin‐1**	2.12 (5.88)	1989.68 (241.82)	8.17 (7.77)	7.62 (3.42)	11.4 (7.07)	67.43 (11.62)	1627
**Coyote Basin‐2**	1.79 (6.53)	1729.71 (243.86)	7.59 (5.93)	8.38 (3.83)	12.92 (7.84)	68 (10.88)	1672
**Wyoming** WTPD							
**Pitchfork Ranch‐1**	0.44 (1.10)	3412.5 (626.96)	14.33 (10.1)	9.53 (1.19)	22.45 (8.44)	3.75 (2.66)	2130
Pitchfork Ranch‐2	0.48 (1.25)	2921.04 (504.49)	8.29 (5.63)	9.53 (1.19)	22.45 (8.44)	3.75 (2.66)	2087
